# Frequency of Convergence Insufficiency Among Young Adults in a University Population

**DOI:** 10.22599/bioj.537

**Published:** 2026-05-19

**Authors:** Nishanee rampersad, Nashka Naidoo, Sibulelo Dzanibe, Husna Alli, Tasneem Makada, Owethu Mthethwa, Tamara Pillay, Minenhle Dube

**Affiliations:** 1 University of KwaZulu-Natal, South Africa

**Keywords:** convergence insufficiency, near point of convergence, heterophoria, fusional vergence, young adults

## Abstract

**Background:**

Convergence insufficiency (CI) is a binocular vision disorder that has been linked to increasing visual demands of near tasks. As young adults routinely perform many near-work tasks, often with digital devices, this study evaluated the frequency of CI among young adults in a university population.

**Method:**

In this cross-sectional study, young adults aged 18–25 years were recruited using convenience sampling. Participants were excluded if they had any ocular pathology, clinically significant anisometropia, strabismus, accommodation insufficiency, reduced visual acuity and history of ocular conditions and vision therapy. Cover test, positive fusional vergence (PFV) and near point of convergence (NPC) were performed. The clinical diagnosis of CI was based on the CI and Reading Study group criteria. Data were analysed using descriptive and inferential statistics.

**Results:**

The mean age of the sample (n = 192) was 21.1 ± 2.0 and consisted of more females (n = 106, 55%). Overall, 151 (79%) participants did not have CI while 41 (21%) had CI. In participants with CI, low suspect CI was most common (n = 26, 63%) followed by high suspect CI (n = 10, 24%) and definite CI (n = 5, 12%). Participants with CI had significantly higher NPC break and recovery values, larger exophoria measurements at distance and near and lower PFV break and recovery values (p < 0.001).

**Conclusion:**

The findings show that CI was present in 21% of young adults in this university population. The test results, for NPC, prism cover test and PFV, in individuals with CI were significantly different from the test results in individuals without CI.

## Introduction

Convergence insufficiency (CI) is a motor disorder characterised by the inability of the eyes to efficiently converge at near (Scheiman and Wick, 2014). Clinical signs of CI include receded near point of convergence (NPC), exophoria (XOP) greater at near than at distance and reduced positive fusional vergence (PFV) at near (Rouse *et al*., 1998). Symptoms of CI include diplopia, asthenopia, difficulty concentrating, blurry vision and headaches when performing near tasks (Scheiman and Wick, 2014).

CI affects people of all ages with a recent review reporting prevalence values ranging from ~2 to 33% (Gantz and Stiebel-Kalish, 2022). In studies involving young adults, the prevalence of CI ranges from 3.4 to 10.2% (Table 1). A study by Obioma-Elemba *et al*. (2021) involving a sample of young adults from Nigeria reported an abnormally higher CI prevalence of 58.6% (Table 1). These broad ranges of prevalence values may be a result of different population characteristics, clinical methods used to assess for CI, cut-off values and/or criteria used to diagnose CI (Gantz and Stiebel-Kalish, 2022; García-Muñoz *et al*., 2016). In a review study, Gantz and Stiebel-Kalish highlighted the considerable variability in clinical diagnostic criteria used in CI prevalence studies over the years (Gantz and Stiebel-Kalish, 2022). The use of varying diagnostic criteria for CI, in terms of the type and number of clinical signs, limits the comparison of prevalence values across studies (Gantz and Stiebel-Kalish, 2022). The criteria from the convergence insufficiency and reading study group (CIRS) have been adopted and used in several studies (Obioma-Elemba *et al*., 2021; Rouse *et al*., 1999).

**Table 1:** Prevalence of CI in studies involving young adults.


AUTHOR (YEAR)	SAMPLE SIZE (n)	MEAN AGE	AGE RANGE (YEARS)	PREVALENCE (%)

Darko-Takyi *et al*. (2022)	300	21.6 ± 2.2	18–30	9.7

Obioma-Elemba *et al*. (2021)	162	NR	18–25	58.6

Horwood, Toor and Riddell, (2014)	167	NR	18–26	10.2

Ma *et al*. (2019)	415	NR	21–38	9.6

García-Muñoz *et al*. (2016)	175	22.9 ± 4.0	18–35	3.4

Porcar and Martinez-Palomera (1997)	65	NR	19–25	7.7


NR, not reported.

The presence of CI has been linked to the increasing visual demands of near-work activities that are sometimes performed for extended periods (Hashemi *et al*., 2017; Von Noorden and Campos, 2002). Contemporary changes in near-work activities due to digital technology may be causing additional stress on the visual system, potentially worsening symptoms of CI (Dehghan, Rafieepour and Hosseinzadeh, 2025). This may be particularly relevant for young adults given their use of their multiple digital devices for academic, communication-related and recreational activities (Obioma-Elemba *et al*., 2021). Recent articles regarding CI have noted that while several studies have investigated CI in the elderly and young children, few studies focus on CI in young adults (Darko-Takyi *et al*., 2022; Gantz and Stiebel-Kalish, 2022). The aim of this study was therefore to evaluate the frequency of CI among young adults in a university population.

## Methods

The study population included young adults aged between 18 and 25 years that were registered at the University of KwaZulu-Natal (UKZN). In this cross-sectional study, participants were recruited using convenience sampling and provided written informed consent after discussion of the nature of the study. A minimum sample of 188 participants was calculated using 5% margin of error and Z score of 1.96 at a 95% confidence interval (Z. Dessie, 2024, personal communication). All participants underwent screening comprising case history (for information on demographics, medical and ocular history), visual acuity at distance and near, cover test at distance and near, refraction (objective and subjective), assessment of accommodation function (amplitude, response and relative measures at a 40 cm test distance and using standardised targets and procedures (Scheiman and Wick, 2014)) and direct ophthalmoscopy. Individuals with visual acuity (unaided or aided) of at least 0.00 LogMAR at 4 m and 1M/0.40 LogMAR at 40 cm, without any ocular pathology, clinically significant anisometropia (difference of 1.00 D or more in either the sphere or cylinder), strabismus, accommodation insufficiency, previous history of ocular injuries, surgeries and vision therapy were included.

Eligible participants proceeded to data collection wherein all procedures were performed with the best refractive correction to ensure test results were not influenced by any uncorrected refractive errors. These procedures included assessment of the NPC, PFV and heterophoria testing. The NPC, which was assessed using the line and dot target as recommended for pre-presbyopes (Rosenfield and Logan, 2009), was measured from the bridge of the nose using a Royal Air Force (RAF) ruler to the break and recovery points (Gantz and Stiebel-Kalish, 2022). The break point measurement was taken when participants either reported sustained diplopia or an outward eye movement was observed (Scheiman and Wick, 2014). The recovery point measurement was taken when the participant reported single vision or when motor fusion was regained (Gantz and Stiebel-Kalish, 2022). The PFV was evaluated using step vergence as this allowed for better visibility of the participant’s eyes (Gantz and Stiebel-Kalish, 2022; Scheiman and Wick, 2014). Negative fusional vergence (NFV) at near was measured only if the participant presented with a near esophoria. The break (sustained diplopia) and recovery (single vision) values were noted for both PFV and NFV (Scheiman and Wick, 2014). The prism cover test, which has been recommended by a recent review (Gantz and Stiebel-Kalish, 2022), was used for objective measurement of the heterophoria at distance and near.

The results for the prism cover test, NPC and PFV were used to classify the presence or absence of CI using the diagnostic criteria of the CIRS group. According to the CIRS group, a clinical diagnosis of CI includes the following signs: (a) near XOP 4^Δ^ than distance phoria, (b) insufficient PFV (failing Sheard’s criterion or a blur value 12^Δ^ or break value 15^Δ^) and (c) receded NPC (break ≥ 7.5 cm or recovery ≥ 10.5 cm) (Rouse *et al*., 1998). Using the CIRS criteria, the four possible diagnostic groups for CI are no CI (non-XOP at near or < 4^Δ^ difference between distance and near XOP), low suspect CI (XOP at near and one sign), high suspect CI (XOP at near and two signs), and definite CI (XOP at near and three signs) (Rouse *et al*., 1998).

A pilot study involving five participants was performed to validate and standardise the data collection procedure. The same environment, instructions and researchers were used to perform each clinical test for standardisation throughout data collection. Standard optometric tests were used for measurement of the NPC, PFV and cover test. Three measurements for NPC, PFV and prism neutralisation cover test were taken and averaged.

The Statistical Package for Social Sciences version 29 was used to capture and analyse the data. A two-step data capture method was used where one researcher entered the data while another researcher validated the accuracy of data entry at a subsequent time. Any discrepancies in this two-step data capture method were corrected prior to data analysis. The data are presented using descriptive statistics such as frequencies, percentages, means, standard deviations and ranges. The Shapiro-Wilk test was used to assess the normality of the clinical measurements and showed that they were asymmetrically distributed. The Mann-Whitney test was used to evaluate the differences between the clinical test results in participants classified with and without CI. A probability (p) value of less than 0.05 was considered statistically significant.

## Results

**Figure 1:**
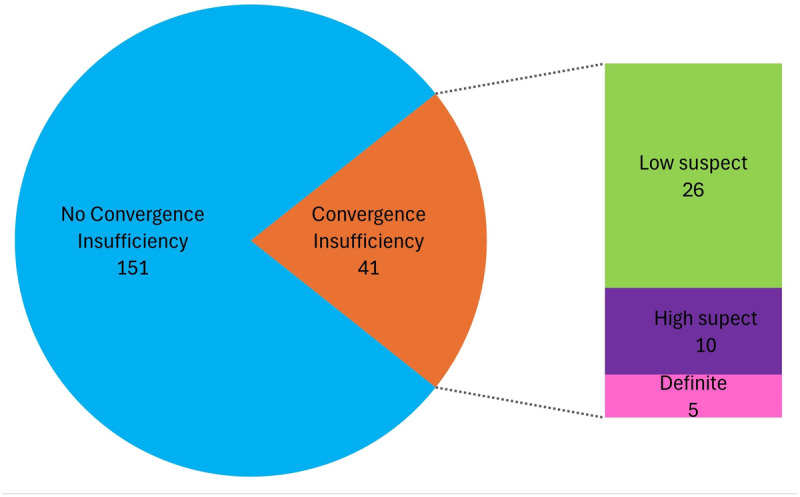
Frequency of CI classification in participants (n = 192).

The sample included 192 participants with a higher proportion of females (n = 106, 55%) than males (n = 86, 45%). The mean age of the sample was 21.1 ± 2.0 years (range, 18–25 years). The mean spherical equivalent refraction for the right and left eyes was –0.72 ± 1.29 D (range, +0.88 to –6.13 D) and –0.79 ± 1.17 D (+0.50 to –6.13 D), respectively. Overall, 41 (21%) participants were classified with CI while 151 (79%) did not have CI. Of those participants with CI, 26 (63%) were classified as low suspect CI, 10 (24%) as high suspect CI and 5 (12%) as definite CI (Figure 1).

Table 2 shows the mean values for the clinical test results in participants classified with (n = 41) and without (n = 151) CI. Participants with CI had significantly higher NPC break and recovery values, larger XOP measurements at distance and near and lower PFV break and recovery values (p < 0.001) (Table 2).

**Table 2:** Comparison of clinical results in participants with and without CI.


CLINICAL TESTS	CI (n = 41)	RANGE	NO CI (n = 151)	RANGE	MEAN DIFFERENCE	p-value*

NPC break (cm)	7.22 ± 3.32	3.00–19.00	4.13 ± 1.60	1.00–8.00	3.09	<0.001

NPC recovery (cm)	9.47 ± 4.26	2.00–25.00	5.94 ± 1.97	1.00–14.00	3.54	<0.001

Heterophoria at distance (^Δ^) Negative and positive values refer to exophoria and esophoria, respectively	–2.95 ± 2.15	–8.00 to –1.00	–0.37 ± 2.69	–7.00 to 6.00	2.58	<0.001

Heterophoria at near (^Δ^) Negative and positive values refer to exophoria and esophoria, respectively	–4.17 ± 2.47	–14.00 to –1.00	–1.32 ± 2.33	–9.00 to 5.00	2.85	<0.001

PFV break (^Δ^)	19.32 ± 11.60	4–45	25.95 ± 10.94	4–45	6.63	<0.001

PFV recovery (^Δ^)	16.00 ± 11.29	1–45	21.96 ± 11.51	1–45	5.96	<0.001


*, Mann-Whitney test. CI, convergence insufficiency; NPC, near point of convergence; cm, centimetre; Δ, prism; PFV, positive fusional vergence.

## Discussion

Considering the ubiquitous use of digital devices for many near-work tasks, it is becoming increasingly important to evaluate ocular disorders associated with close working distances. Approximately 90% of individuals use digital devices for two hours or more each day, with 70% using multiple devices simultaneously (The Vision Council, 2016). These devices are being used by individuals of all age groups and for various everyday activities (The Vision Council, 2016). The relationship between ocular disorders and near-work performance—manifesting as decreased academic achievement, near-work fatigue, problems with reading and reduced quality of life—is well recognised (Hashemi *et al*., 2017; Hussaindeen and Murali, 2020; Scheiman and Wick, 2014). This study reports on the frequency of CI among young adults in a university population. These results may be relevant given the increasing demands of modern-day near-work tasks and limited research on CI in young adults, especially in Africa. In this study, approximately one out of every five individuals was classified with CI. This finding highlights that CI is a relatively common non-strabismic vergence disorder among young adults, corroborating results from previous studies (García-Muñoz *et al*., 2016; Hashemi *et al*., 2017; Ma *et al*., 2019; Ovenseri-Ogbomo and Ovigwe, 2016).

In the present study, 21% of participants were classified with CI when using the CIRS group diagnostic criteria to classify CI. In contrast, Obioma-Elemba *et al*. (2021) reported a much higher prevalence value, where 59% of their sample of university students had CI despite using the same CIRS group CI criteria (Obioma-Elemba *et al*., 2021). The notably higher proportion of participants with CI in the study by Obioma-Elemba *et al*. (2021) must be interpreted with caution as the von Graefe test, which lacks repeatability and shows poor agreement with the gold standard cover test (Elliot, 2025), was used to measure the heterophoria. Furthermore, a pen tip was used as the fixation target for the NPC test by Obioma-Elemba *et al*. (2021) and this may also account for the difference in prevalence value compared with the present study. Other studies including young adult samples (Darko-Takyi *et al*., 2022; Horwood, Toor and Riddell, 2014; Ma *et al*., 2019) reported that a lower percentage (~10%) of their participants had CI and this difference is likely explained by the use of varying criteria, clinical tests and cut-off values for these clinical tests to diagnose CI. It is also possible that differences in sample characteristics, in terms of size, age range and gender distribution, account for this variability in findings.

The CIRS group criteria enable individuals diagnosed with CI to be further sub-classified into three groups corresponding to the severity of the CI clinical signs. Of the participants diagnosed with CI in this study (n = 41), the most common diagnostic group was low suspect (63%) followed by high suspect (24%) and definite (12%) CI. This trend is consistent with the results of previous studies (Obioma-Elemba *et al*., 2021; Rouse *et al*., 1998) where the frequency of low suspect CI was highest while definite CI was lowest. For example, in an early study Rouse *et al*. (1998) reported that of the 209 children diagnosed with CI, 136 (65%), 48 (23%) and 25 (12%) were classified as low suspect, high suspect and definite CI, respectively. The value of knowing the CI subgroup helps eye care personnel evaluate the severity of the CI diagnosis at baseline and could be useful to monitor the effectiveness of the CI management. In patients undergoing vision therapy for CI, the decrease in the number of clinical signs of CI could be considered in conjunction with the patient’s subjective report of symptoms experienced when performing near-work tasks to holistically understand the objective and subjective impact of the CI treatment.

As expected, the clinical test results in participants classified with CI were more deviated from the expected range of values compared with participants without CI. Overall, participants classified with CI had higher NPC break and recovery values, higher distance and near XOP measurements and lower PFV break and recovery values. These findings are consistent with previous studies that also compared clinical test results in individuals with and without a clinical diagnosis of CI (Hashemi *et al*., 2017; Obioma-Elemba *et al*., 2021; Rouse *et al*., 1998).

Symptoms of untreated ocular disorders such as CI have the potential to influence performance of near-work tasks and consequently cause academic problems and reduce quality of life (Hashemi *et al*., 2017; Hussaindeen and Murali, 2020). There is therefore a need for early identification and management of CI to minimise its impact on the performance of near-work tasks. Eye care personnel should give more attention to near-work symptoms and consider using validated questionnaires such as the Visual Discomfort Scale (Conlon *et al*., 1999), CI Symptom Survey (Borsting, Rouse and De Land, 1999) and College of Optometrists in Vision Development Quality of Life Outcomes Assessment (Maples, 2000) to investigate these symptoms, particularly in young adults. The findings in the present study emphasise the need for greater awareness and screening activities related to ocular disorders, especially CI, in student populations. Vision screening activities should therefore be routinely undertaken in these populations given the impact symptoms of CI can have on near-work activities and academic outcomes (Hashemi *et al*., 2017). Early identification and management have the potential to minimise associated near-work symptoms, improve near-work performance and allow for optimal academic achievement in these young adult university students.

The study has several limitations. Firstly, the study did not investigate the association of refractive error on the clinical diagnosis of CI and this should be included in future studies. Although this study adds to knowledge of CI from an African perspective, the sample was limited to young adults from one higher education institution. This implies that the study results may not be generalised to other university populations. Future studies involving South African populations with a wider age range in different settings—such as schools, old-age homes, other higher education institutions—should therefore be conducted. Furthermore, the study used convenience sampling limiting the generalisability of the study findings. It is therefore recommended that future studies consider probability sampling methods to minimise potential selection bias. Lastly, this study did not report on the symptoms of CI as it was beyond the scope of the current investigation. It is therefore recommended that future studies investigate both the signs and symptoms of CI.

## Conclusion

The study reveals that CI is a relatively common ocular disorder and was present in 21% of young adults in a university population in the present study. Participants with CI had significantly higher NPC break and recovery values, larger XOP measurements at distance and near and lower PFV break and recovery values than those without CI.
